# Fetal brain response to worsening acidosis: an experimental study in a fetal sheep model of umbilical cord occlusions

**DOI:** 10.1038/s41598-023-49495-2

**Published:** 2023-12-27

**Authors:** Laure Lacan, Charles Garabedian, Julien De Jonckheere, Louise Ghesquiere, Laurent Storme, Dyuti Sharma, Sylvie Nguyen The Tich

**Affiliations:** 1 CHU Lille, Univ. Lille, ULR 2694 - METRICS, 59000 Lille, France; 2 Department of Pediatric Neurology, CHU Lille, 59000 Lille, France; 3 Department of Pediatric Neurology, Hôpital Roger Salengro, CHU Lille, Avenue du Professeur Emile Laine, 59037 Lille Cedex, France; 4 Department of Obstetrics, CHU Lille, 59000 Lille, France; 5 CHU Lille, CIC-IT 1403, 59000 Lille, France; 6 Department of Neonatology, CHU Lille, 59000 Lille, France; 7 Department of Pediatric Surgery, CHU Lille, 59000 Lille, France

**Keywords:** Neuroscience, Neurology

## Abstract

Perinatal anoxia remains an important public health problem as it can lead to hypoxic–ischaemic encephalopathy (HIE) and cause significant neonatal mortality and morbidity. The mechanisms of the fetal brain’s response to hypoxia are still unclear and current methods of in utero HIE prediction are not reliable. In this study, we directly analysed the brain response to hypoxia in fetal sheep using in utero EEG. Near-term fetal sheep were subjected to progressive hypoxia induced by repeated umbilical cord occlusions (UCO) at increasing frequency. EEG changes during and between UCO were analysed visually and quantitatively, and related with gasometric and haemodynamic data. EEG signal was suppressed during occlusions and progressively slowed between occlusions with the increasing severity of the occlusions. Per-occlusion EEG suppression correlated with per-occlusion bradycardia and increased blood pressure, whereas EEG slowing and amplitude decreases correlated with arterial hypotension and respiratory acidosis. The suppression of the EEG signal during cord occlusion, in parallel with cardiovascular adaptation could correspond to a rapid cerebral adaptation mechanism that may have a neuroprotective role. The progressive alteration of the signal with the severity of the occlusions would rather reflect the cerebral hypoperfusion due to the failure of the cardiovascular adaptation mechanisms.

## Introduction

Perinatal anoxia still represent 1 to 5 per 1000 full-term deliveries in western countries^1–4^. Severe anoxia can lead to hypoxic-ischemic encephalopathy (HIE), which can be fatal for the child or cause long-term neurological disability. Nevertheless, neurophysiopathological mechanisms of fetal cerebral anoxia remain partially known and the current methods of intrauterine detection of HIE risk (fetal heart rate analysis, fetal blood sample, ST analysis) are unreliable^5,6^.

Animal models are currently essential to evaluate the fetal brain direct response to anoxia during labor and to develop new methods for early detection and prevention of neonatal HIE. The ovine model is particularly relevant because their fetal physiology is similar to Humans, especially at the hemodynamic and cerebral level^7^. Brain maturation in the fetal sheep is faster than in humans but follows same ontogenic pattern of evolution with gestational age^7–10^. Several fetal sheep experimental models of perinatal anoxia were previously published with different protocols of induced anoxia such as bilateral carotid occlusions^11,12^ or umbilical cord occlusions (UCO) with variable duration and frequency^13–16^. UCO is a partial reflection of intra uterine contraction with a reduction of flow intrauterine as during umbilical cord compression, one of the most common causes of perinatal anoxia in Humans^17^. Previous studies mostly analysed the effects of prolonged total cord occlusion in the fetus. In clinical practice this situation corresponds to severe cord procidence, can be easily detected, and rarely represents a therapeutic dilemma.

Repeated intermittent cord occlusion protocols of increasing severity, as may occur during maternal labour, have been shown to result in progressive acidosis, haemodynamic changes and heart rate variability abnormalities^18–22^. However, because of the initial efficiency of the fetal cardiovascular and cerebral adaptation mechanisms to anoxia, the depth of acidosis in particular does not reflect well the fetal cerebral impact of anoxia, and appears to be a poor predictor of ischemic brain damage and long term outcome^6,23–26^. Direct study of the fetal brain to analyse its response to anoxia in real time therefore seems crucial, in order to detect brain damage as early as possible.

Therefore, the objective of this study was to assess the fetal sheep cerebral response to progressive hypoxia using EEG visual and quantitative analysis in our model of UCO.

## Methods

### Ethics

The anesthesia, surgical and experimental protocols followed the recommendations of the National Institutes of Health guide for the care and use of Laboratory animals (NIH Publications No. 8023, revised 1978) and the ARRIVE guidelines. The study was approved by the Animal Experimentation Ethics Committee of “Hauts-de-France” (CEEA #2016121312148878).

### Surgical procedure

Near term fetal sheep (124 ± 1 days gestational age, term: 140 days, breed ‘Ile de France’, coming from Tours’INRA, Orfrasière animal physiology experimental Unit, Val de Loire Center) were surgically instrumented following the protocol detailed in our previous publications^21,27–29^.

After a 24-h fast, the ewes were operated under general anaesthesia, induced by inhaled 5% Isoflurane (Isoflurane, Aerrane®, Baxter, Maurepas, France) after premedication with Xylazine 0.3 mL/kg (Sedaxylan® 20 mg/mL, Dechra, Netherlands). Sedation was maintained by inhaled Isoflurane 2% and maternal analgesia was provided by Buprenorphine 0.6ml/10kg (Bupaq®, Virbac, France). After maternal midline laparotomy and hysterotomy under surgical aseptic conditions, two 4Fr diameter catheters (Arrow, USA) were inserted into both fetal axillary arteries. Four precordial electrodes (MYWIRE 10, MAQUET, Germany) were placed on intercostal muscles for ECG signal recording.

For EEG placement, we have recently developed a new EEG recording technique using electrodes placed in utero under the scalp of the foetus, providing a real-time recording of its brain activity^27^. The fetal head was exposed through the hysterotomy. Four needle electrodes (MYWIRE 10, MAQUET, Germany) were fixed on both sides over frontal and parietal cortex, 10 mm lateral to the sagittal suture and 5 mm anterior to the fronto-parietal suture. A reference electrode and a ground electrode were placed over the occiput. Each electrode was placed under the cranial periosteum through 5 mm skin incision, in contact with the bone to completely isolate it from the amniotic fluid. The electrode was attached to the scalp suture (Vicryl 2/0 Rapide). An inflatable silicone occluder (OC VO-16HD—DOCXS Biomedical Products—Ukiah, California) was placed around the fetal umbilical cord.

The placental circulation was maintained intact and the fetus was placed back into the uterus after instillation of 500 mg of amoxicillin-clavulanic acid (Amoxicillin-clavulanic acid SANDOZ®, Sandoz, Levallois-Perret, France) into the amniotic fluid. The occluder, catheters, EEG and ECG wires connected to extenders were externalized on the right lateral flank of the sheep by subcutaneous tunnelling.

After the operation, the permeability of the fetal catheters was maintained by a daily injection of 10 IU/mL heparinised isotonic saline (Heparin CHOAY®5000IU, Sanofi-aventis France, Paris). A maternal intramuscular injection of 0.3 mL/10 kg of Buprenorphine (Bupaq®, Virbac, France) was performed 24 and 48 h after the operation to ensure postoperative analgesia. Antibiotic prophylaxis was performed by a daily injection of 1 mL/kg of Amoxicillin intramuscularly (Clamoxyl LA® 150 mg/mL, Zoetis, France) during the 3 days of postoperative rest.

### Experimental procedure

**Figure 1:**
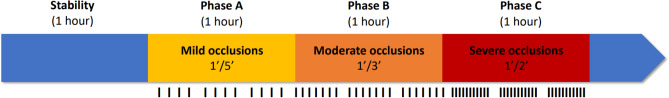
Experimental protocol. At day 4 after surgery: progressive anoxia induced by repeated umbilical cord occlusions of increasing frequency.

The experimental protocol started on the fourth day after surgery. After a stability period of one hour, anoxia was induced by repeated umbilical cord occlusions of increasing frequency, as previously described^17,20,30^. Three phases of one hour each were performed successively, with 1-min occlusions repeated every 5 min during phase A (mild occlusions), every 3 min during phase B (moderate occlusions) and every 2 min during phase C (severe occlusions). Every 20 min a 5-min period without occlusion was observed, called “pause” (Fig. 1). The protocol was stopped if the arterial pH fell below 6.90.

After the protocol, animals were sacrificed by a maternal intravenous injection of 6 mL/50 kg of T61® (Embutramide 20g, Mebezonium iodide 5g, Tetracaine hydrochloride 0.5g, Dimethylformamide 60ml).

### Data acquisition

Fetal EEG signal and haemodynamic data were recorded continuously during the stability and occlusion phases.

The EEG signal from each channel was recorded monopolar against the reference electrode using EEG System plus Evolution software (Micromed SAS, Macon, France), filtered between 0.5 and 70 Hz and digitised at a sampling rate of 256 Hz. The signal was analysed before the occlusions during the stability phase (1 h), per-occlusion during the last occlusion of each phase (1 min) and during the whole duration of each occlusion phase (1 h).

The fetal heart rate was recorded by the 4 precordial ECG electrodes and the arterial pressure monitored by the fetal arterial catheters connected to pressure sensors (Pressure Monitoring Kit, Baxter, France). These haemodynamic data were visualised on a multiparametric anaesthesia-resuscitation monitor (Merlin monitor, Helwett Packard, Palo Alto, CA, USA) and recorded on computer media using Physiotrace® software. Heart rate (HR) and mean blood pressure (MBP) were analysed before the occlusions, during the stability phase (1 h), per-occlusion during the last occlusion of each phase (1 min) and between the occlusions during the last pause of each phase (5 min).

Gasometric data (pH, pO2, pCO2, lactate) were measured from arterial blood samples using the I-STAT® micro-method (Abbott Laboratories, Abbott Park, IL USA). Measures were taken in the stability phase and during the last pause of each occlusion phase.

### EEG analysis

The entire EEG recordings were visually analysed for frequencies, amplitude and lability by two examiners (LL and SN) trained in neonatal EEG analysis using System Plus Evolution software (Micromed SAS, Macon, France). The EEG was displayed in bipolar montage on 30-s pages (filter 0.5–70 Hz, gain 70 µV)*.*

The raw EEG signal was analysed quantitatively using three qEEG features available in the program “EEG Analyser” (Micromed®): the minimum Amplitude Index (min AI), the Burst Suppression Ratio (BSR) and the Spectral Edge Frequency (SEF). These features were measured on 2-s intervals on frontal and parietal channels in bipolar montage and then averaged over the two channels.

Min AI was calculated as the minimum peak of the EEG amplitude envelope (CFM) over the analysed interval (µV), after filtering the signal at 2–20 Hz. BSR was measured as the ratio of signal suppression, detected for an amplitude < 10 µV during > 500 ms, on the total length of the analysed interval (%). SEF was defined as the frequency (Hz) below which 95% of the power in EEG exists in the analysed interval.

Median values of each qEEG and haemodynamic feature were calculated during the stability phase (1 h pre-occlusion), during the last occlusion of each phase (1 min) to analyse immediate response to anoxia, and during the whole duration of each phase (1 h of mild, moderate and severe occlusions) to analyse the progressive brain adaptation to repeated anoxia.

### Statistical analyses

Gasometric, haemodynamic and qEEG data were described by the median (1st–3rd quartile) for each phase studied.

The difference between the stability phase and the three occlusions phases A, B and C was analysed by a Friedman test for repeated measures (significance level p ≤ 0.05). When the difference was significant, a post-hoc analysis comparing the different phases 2 by 2 was performed by a Wilcoxon test (significance threshold p ≤ 0.05).

The correlation between the qEEG and the different gasometric and haemodynamic markers was analysed by a Spearman test (significance level p ≤ 0.05).

These statistical analyses were performed with XlStat software for Microsoft Excel (AddInSoft, Paris, France) and with SPSS version 20.0 software (SPSS, IBM).

## Results

Fourteen pregnant ewes were instrumented. Two fetal deaths occurred in utero at postoperative day 1, one delivery occurred at postoperative day 3, one occluder ruptured during the first phase of UCO and one foetus was excluded because it presented severe cerebral lesions whose origin appeared to be prior to the protocol. A total of 9 fetuses were included for analysis.

### Data evolution with progressive hypoxia

The pH decreased progressively during the experimental procedure (Table 1), reaching a severe acidosis (pH 6.98) in phase C. PCO2 and lactate increased between each phase (p < 0.001), while PO2 remained stable (p = 0.430). Heart rate during pauses between occlusions remained stable over the different phases (p = 0.736). A per-occlusion bradycardia was recorded from the very first occlusion in phase A (85 bpm versus 178 bpm) and then remained relatively stable throughout the three occlusions phases. Mean blood pressure measured during pauses between occlusions increased between stability phase and phases B and C (p = 0.037). Per-occlusion mean blood pressure decreased significantly in phase C compared to A and B (p = 0.003).

**Table 1:** Gasometric and haemodynamic data according to stability and occlusion phases (N = 9).

	Stability	Occlusion phases			
	S (n = 9)	A (n = 9)	B (n = 9)	C (n = 8)	p
pH	7.39 (7.37; 7.41)	**7.28 (7.23; 7.35)**^**x**^	**7.10 (7.07; 7.27)**^x^*	**6.98 (6.84; 7.08)**^x^*^+^	** < 0.001**
PO2 (mmHg)	12 (9.5; 19)	16.0 (11.5; 17.0)	15.0 (13.0; 17.5)	16.5 (13.5; 18.8)	0.430
PCO2 (mmHg)	45.3 (43; 49.5)	**50.6 (48.5; 56.2)**^**x**^	**54.0 (52.5; 63.1)**^x^*	**64.9 (55.1; 87.0)**^x^*^+^	** < 0.001**
Lactate (mmol/l)	2.27 (1.69; 3.16)	**6.0 (3.4; 8.3)**^**x**^	**12.8 (7.3; 15.3)**^x^*	**15.9 (13.6; 16.3)**^x^*^+^	** < 0.001**
pHR (bpm)	186 (175; 193)	178 (151; 187)	189 (158; 202)	175 (150; 192)	0.736
pMBP (mmHg)	48 (42; 51)	58 (47; 65)	**62 (51; 66)**^**x**^	**64 (54; 68)**^**x**^	**0.037**
ocHR (bpm)	–	**85 (72; 96)**^**x**^	**80(69; 95)**^**x**^	**79 (54; 92)**^**x**^	**0.001**
ocMBP (mmHg)	–	56 (51; 62)	54 (43; 62)	**37 (20; 50)**^x^*^+^	**0.003**

Median values (1st–3rd quartile) calculated pre-occlusion in stability phase (S); between occlusions during the last pause of each phase (A,B,C) for gasometric data, pause heart rate (pHR) and pause mean blood pressure (pMBP); per-occlusion during the last occlusion of each phase for per-occlusion heart rate (ocHR) and per-occlusion mean blood pressure (ocMBP).

The p-values are calculated by a Friedman test between the stability phase and the three occlusions phases.

Wilcoxon test: ^x^significant values versus Stability; *significant values versus Phase A; ^+^significant values versus Phase B (p < 0.05).

Significant values are in bold.

EEG visual analysis showed a continuous, mild amplitude and labile signal during the stability phase (pre-occlusion), as described in our previous publication^27^.

During the phase A (mild occlusion) there was few visual EEG changes during and between occlusion.

**Figure 2:**
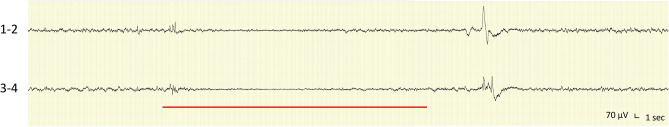
Example of fetal sheep EEG tracing during cord occlusion. A diminution of the signal amplitude can be seen during this UCO in phase B (1 min of occlusion every 3 min)—90 s epoch. The red bar mark the occlusion. Transverse bipolar montage with derivations 1–2 corresponding to left and right frontal electrodes and 3–4 to left and right parietal electrodes.

In phase C (severe occlusions), occlusions visually resulted in immediate EEG changes with a rapid decrease in amplitude or even a complete suppression of activity, sometimes preceded by transient large waves (Fig. 2). The release of occlusion was followed by high amplitude transient waves and then a rapid recovery of the activity. Between occlusions the EEG signal was characterised by low amplitudes and slower waves than in the previous phases.

**Figure 3:**
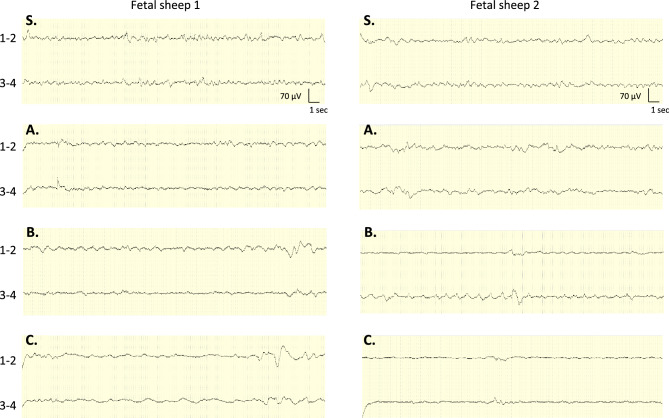
Two examples of fetal sheep EEG tracings before and during cord occlusions. Extracts pre-occlusion in stability phase (S) and between occlusions in mild phase (A), moderate phase (B) and severe phase (C)—30 s epochs. EEG signal alteration can be seen in both fetuses from moderate occlusions phase with slowing and decrease in amplitude. Transverse bipolar montage with derivations 1–2 corresponding to left and right frontal electrodes and 3–4 to left and right parietal electrodes.

**Figure 4:**
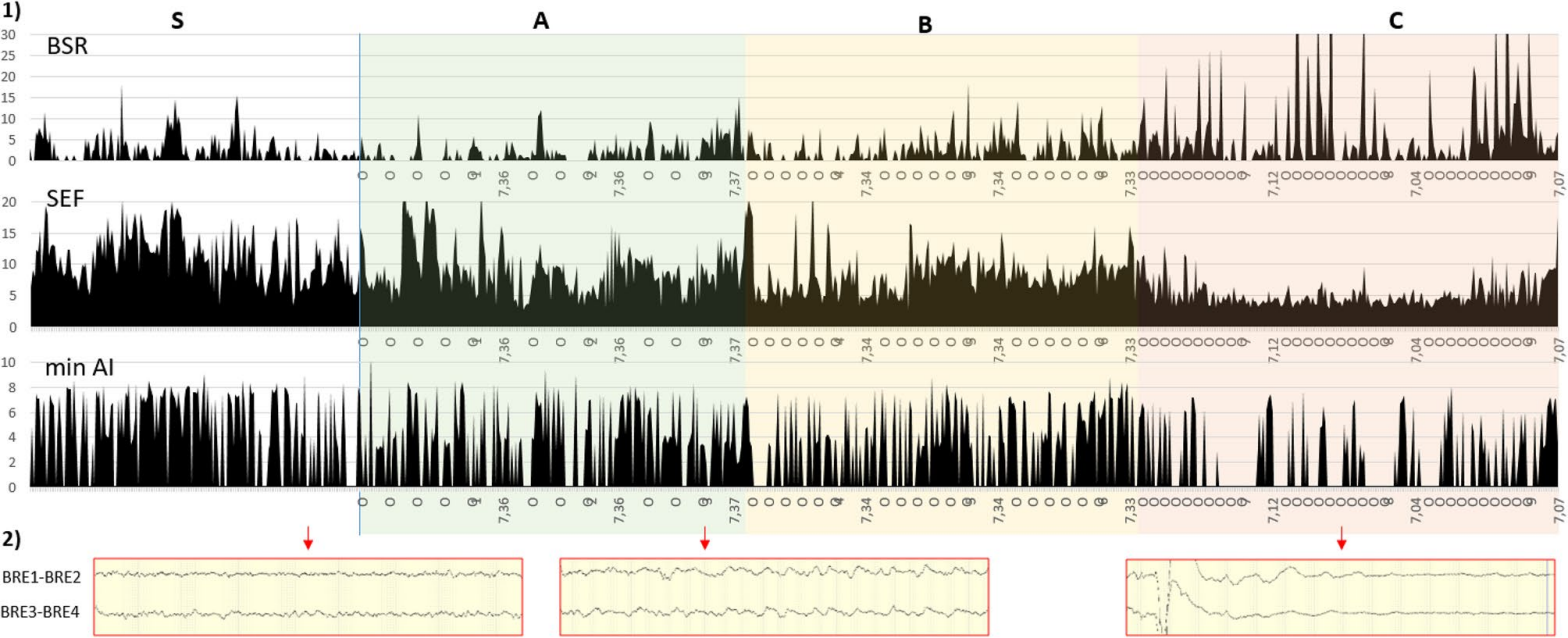
Example of qEEG curves in a fetus in stability phase and during the protocol of progressive hypoxia. (**1**) Evolution of BSR (%), SEF (Hz) and minAI (µV) values in stability phase (S), in mild occlusions phase (A), moderate occlusions phase (B) and severe occlusions phase (C), of 1 h each in fetal sheep 3. O = total cord occlusion (1 min). (**2**) 30-s EEG extracts in stability phase and at the end of a mild and severe occlusion.

In phase B, EEG changes varied between fetuses but some modifications could be observed from the beginning of that phase during and between occlusions (Figs. 3, 4).

Quantitative analysis (Table 2) during stability phase (pre-occlusion) showed that BSR median ranged from 0 to 7% except for one foetus where the median was 26%. The SEF ranged from 5.3 to 16.3 Hz and the minimum amplitude from 0.1 to 7.0 µV. The SEF values fluctuated during the stability phase (Fig. 4), corresponding to the physiological variations observed visually.

**Table 2:** qEEG data according to stability and occlusion phases (N = 9).

	Stability	Occlusion phases			
	S (n = 9)	A (n = 9)	B (n = 9)	C (n = 8)	p
(1) Whole phase EEG					
wBSR (%)	2.19 (0.34; 6.66)	3.58 (1.29; 7.50)	**8.93 (0.87; 19.19)**^x^*	**6.46 (3.55; 13.96)**^x^*	**0.029**
wSEF (Hz)	6.32 (5.59; 12.26)	7.08 (6.05; 10.19)	6.00 (4.67; 9.57)*	**4.72 (4.39; 9.61)**^x^*	**0.003**
wminAI (µV)	3.79 (1.45; 5.86)	3.73 (1.48; 4.85)	2.20 (0.10; 3.69)	1.01 (0.10; 2.92)	0.066
(2) Per-occlusion EEG					
ocBSR (%)	–	5.03 (1.34; 14.74)	5.07 (1.47; 45.27)	**18.11 (7.72; 25.5)**^**x**^	**0.018**
ocSEF (Hz)	–	7.90 (4.84; 10.63)	6.17 (4.56; 12.83)	**4.44 (3.50; 6.60)**^x^*	**0.008**
ocminAI (µV)	–	3.38 (0.10; 5.56)	3.37 (1.25; 5.3)	0.10 (0.10; 2.86)	0.096

Median values (1st–3rd quartile) calculated in stability phase (S) and during each whole occlusion phase (1); per-occlusion during the last occlusion of each phase (2).

*minAI* minimum amplitude index (µV), *BSR* burst suppression ratio (%), *SEF* spectral edge frequency (Hz): calculated during the occlusion (occ) and globally during the entire phases (global).

The p-values are calculated by a Friedman test between the stability phase and the three occlusions phases.

Wilcoxon test: ^x^significant values versus stability; *significant values versus Phase A; ^+^significant values versus Phase B (p < 0.05).

Significant values are in bold.

**Figure 5:**
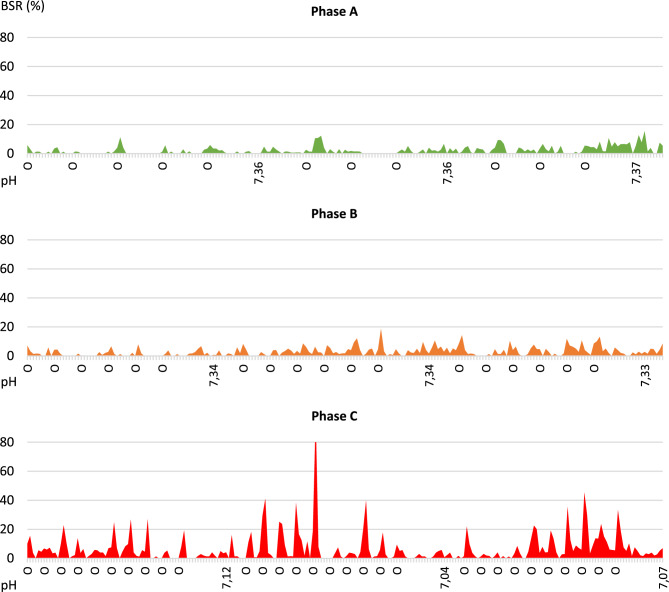
Example of BSR curve during the three occlusions phases of increasing severity. Phase A: mild occlusions (1ʹ/5ʹ). Phase B: moderate occlusions (1ʹ/3ʹ). Phase C: severe occlusions (1ʹ/2ʹ). O = onset of cord occlusion (1 min). pH on the x-axis.

During occlusions, the BSR (ocBSR) increased with a peak depending on the severity of the occlusion (Fig. 5) with marked inter-individual variations (Table 2). Some fetuses presented peaks of BSR during occlusion from phase A, some only in phase B or C. Compared to stability data, the BSR increase was only significant in phase C (p = 0.018) (Table 2(2)). Per-occlusion SEF (ocSEF) decreased significantly in phase C (p = 0.008) and minimum AI (ocminAI) decreased in phase C as well but was not significant (p = 0.096).

During the length of each phase, BSR levels (wBSR) increased progressively between phases, especially between phases A and B (p = 0.029) (Table 2(1)). The SEF significantly decreased in phase B compared to phase A (p = 0.003). The amplitude decreased too with each severity phases but not significantly (p = 0.066).

### Relation between qEEG data and gasometric/haemodynamic data

Some qEEG and gasometric/haemodynamic data were significantly correlated (Table 3). Per-occlusion BSR increase correlated with per-occlusion HR decrease (p = 0.007), pause HR decrease (0.043) and pause MBP increase (p = 0.037).

**Table 3:** Correlation between qEEG and gasometric/haemodynamic data.

Markers	Haemodynamic				Gasometric			
qEEG	pHR (bpm)	pMBP (mmHg)	ocHR (bpm)	ocMBP (mmHg)	pH	PCO2 (mmHg)	PO2 (mmHg)	Lactate (mmol/L)
ocBSR (%)	− **0.400; 0.043**	**0.411; 0.037**	− **0.515; 0.007**	0.151; 0.493	− 0.202; 0.323	0.013; 0.948	0.231; 0.256	0.253; 0.213
ocSEF (Hz)	− 0.167; 0.414	0.034; 0.870	0.104; 0.613	**0.428; 0.041**	**0.480; 0.013**	− **0.434; 0.027**	0.079; 0.700	− **0.416; 0.034**
ocminAI (µV)	− 0.067; 0.746	− 0.042; 0.838	− 0.169; 0.408	**0.572; 0.003**	**0.461; 0.018**	− **0.670; < 0.0001**	− 0.015; 0.943	− 0.316; 0.116
wBSR (%)	− 0.202; 0.322	**0.557; 0.003**	− 0.114; 0.578	0.130; 0.555	− 0.258; 0.203	0.220; 0.280	− 0.008; 0.970	0.284; 0.159
wSEF (Hz)	− 0.163; 0.428	0.058; 0.780	− 0.078; 0.706	**0.637; 0.001**	0.351; 0.078	− **0.629; 0.001**	− 0.135; 0.511	− 0.231; 0.256
wminAI (µV)	− **0.460; 0.018**	0.199; 0.329	0.087; 0.673	**0.579; 0.004**	**0.604; 0.001**	− **0.398; 0.044**	0.159; 0.438	− **0.532; 0.005**

*HR* heart rate, *MBP* mean blood pressure calculated during the pause (p) and during the occlusions (oc), *minAI* minimum amplitude index (µV), *BSR* burst suppression ratio (%), *SEF* spectral edge frequency (Hz): calculated during the occlusion (oc) and during the whole phases (w).

Spearman’s correlation coefficient (r) and p-values.

In bold: significant values (p < 0.05).

Per-occlusion and whole phase decrease in SEF and amplitude correlated with per-occlusion decrease of MBP (respectively p = 0.041 and p = 0.001 for the SEF; p = 0.003 and p = 0.004 for min AI) and with acidosis and increased PCO2. Only the amplitude decrease correlated with the lactate increase (p = 0.034).

BSR did not correlate with biological markers and no qEEG marker correlated with PO2.

## Discussion

In this study we investigated in utero fetal EEG response to progressive hypoxia, caused by repeated UCO of increasing frequency. We observed that EEG signal was suppressed during occlusions and progressively slowed between occlusions as the anoxia protocol progressed. Per-occlusion EEG suppression correlated with per-occlusion bradycardia and increased blood pressure, whereas EEG slowing and amplitude decreases correlated with arterial hypotension and respiratory acidosis.

Amplitude decrease or EEG signal suppression was visually identifiable from the mild occlusions phase in some fetuses and evident for severe occlusions. This signal suppression was quantitatively reflected by a peak of BSR (ratio of signal suppression) and a decrease in SEF (due to the decrease in frequency and amplitude of the signal) with statistically significant modification only during severe occlusions. Quantitative amplitude decreases were not significant, but the amplitude could have been affected by the large waves often occurring at the onset and at the end of the cord occlusion.

Alterations of EEG background could be observed visually from the moderate occlusions phase, with a progressive slowing down of the signal, which no longer normalized between severe occlusions. This was quantified by the decrease of the whole phase SEF, especially in the severe phase. The whole phase amplitude also tended to decrease, but the difference was not significant. The increase in BSR from the moderate occlusion phase onwards was probably related to the closer timing of the occlusions, responsible for more intense and frequent concomitant signal suppression; the signal did not appear to be discontinuous between occlusions.

Our results are comparable with those obtained from ECOGs recordings during experimental anoxia protocols in lamb foetuses. In prolonged anoxia protocols (bilateral carotid occlusion or complete cord occlusion lasting more than 5 min), EEG signal suppression was observed immediately or between 30 and 90 s after occlusion depending on the study^14,16,31^, accompanied by a decrease in mean amplitude, SEF and total power^12,32–34^. For example, in Pulgar’s 2007 study in 120-day gestation fetuses, SEF decreased from a mean of 13.6 ± 1.6 Hz to 10.6 ± 0.8 Hz during 5-min total cord occlusions^35^. Cerebral signal flattening and decrease in SEF concomitant with occlusions and dependent on their severity have also been described in studies using brief repeated cord occlusion protocols^15,22,36^. In Frasch 2011 study, using the same protocol as ours in fetuses of identical term, the mean ECOG amplitude decreased from 88 ± 13 µV in stability to 90 ± 27 µV per mild occlusion, 81 ± 21 µV per moderate occlusion and 60 ± 12µV per severe occlusion. Similarly, the SEF decreased from 14.4 ± 0.4 Hz in stability to 14.1 ± 2.1 Hz per mild occlusion, 11.2 ± 0.9 Hz per moderate occlusion and 9.9 ± 1.1 per severe occlusion. Only the severe per-occlusion values were significantly lower than the stability values and recovery was rapid between occlusions^15^. A progressive decrease in SEF was also found between occlusions of increasing severity in these studies^15,22,37^.

At the end of severe occlusions, Frasch et al. reports a pattern of EEG-ECG synchronisation with a decrease in amplitude and a peak in SEF concomitant with bradycardia and correlated with the onset of arterial hypotension, approximately 1 h before the onset of severe acidosis^15,22,38^. The authors suggest that this pattern may be related to a relative increase in fast frequencies due to the predominance of the activity of inhibitory GABA interneurons capable of high-frequency oscillations^15^. In our opinion, the SEF peak around 25 Hz could be directly related to the signal suppression. In fact, in a recent study analysing the different parameters of the BIS, Connor shows that when EEG is perfectly isoelectric, the signal power is close to zero in all frequency bands, the SEF becomes then undefined and the algorithm returns an unusual value of 30 Hz^39^. Nevertheless, we did not find a peak in SEF concomitant with severe occlusions in the quantitative analysis of our tracings.

Considering the amplitude signal, the results are controversial: De Haan et al. showed a progressive decrease in ECOG amplitude during occlusions (1ʹ/2.5ʹ in fetuses of 126 days gestation) whereas Frasch et al. found no modification or even an increase in the global amplitude with the severity of the occlusion phases. This last result could be linked to a disturbance in the sleep–wake cycles of the foetus. Indeed, some studies suggest that repeated occlusions may result in an increase in large amplitude slow activity^15,31,40^.

The increase in BSR during occlusions correlated with per-occlusion bradycardia and increased blood pressure between occlusions. These haemodynamic changes correspond to cardiovascular mechanisms of adaptation to anoxia previously described in the lamb foetus^41,42^, allowing the redistribution of blood flow to the central organs and the initial maintenance of cerebral perfusion.

Therefore, per-occlusion EEG suppression could reflect a rapid active adaptive neuroprotective mechanism that participates with cardiovascular adaptation in the maintenance of cerebral energy requirement. Indeed, previous experimental animal studies suggest that during acute anoxia, synaptic transmission is rapidly inhibited via inhibitory neuromodulators^43–46^. This extinction of brain activity seems to reduce the energy consumed by the potassium-sodium ionic transport that accompanies the generation of synaptic potentials, and thus the brain metabolism^43–46^, although the precise mechanisms of these phenomena remain to be elucidated. In parallel, the cerebral autoregulatory response to acute hypoxia appears to depend on cardiovascular adaptation mechanisms, which would allow a central redistribution of blood flow through peripheral vasoconstriction and arterial hypertension, and thus the maintenance of cerebral perfusion in the first instance. Moreover, the intensity of this cerebral “shut down” seems directly related to the severity of anoxia since the EEG modification is more pronounced during severe occlusions. Interestingly, the different fetuses did not adapt in the same way since this per-occlusion modification was found more or less early in the experimental procedure.

Otherwise, the progressive alteration of the EEG background (slowing down and tendancy for amplitude to decrease) occurring from moderate occlusions phase was correlated with acidosis and arterial hypotension. This signal alteration could therefore be associated with the failure of cardiovascular adaptation mechanisms. It has been shown that tissue hypoxia leads to progressive acidosis and then cardiovascular failure, with impaired cardiac function and loss of initial peripheral vasoconstriction resulting in arterial hypotension and then cerebral hypoperfusion^47–51^. Yumoto et al. report a decrease in myocardial contractility as soon as the pH drops below 7.20^50^.

During 4-min occlusions repeated every 90 min in lamb fetuses, Kaneko et al. show an increase in cerebral blood flow and perfusion pressure during the first occlusions whereas the pressure increases less or even decreases at the end of the occlusion when occlusions are repeated^16^. The global signal alteration could therefore reflect a more intense cerebral adaptive response due to the cerebral hypoperfusion or already correspond to an anoxic cerebral depolarisation. This would indicate that the autoregulation threshold has been overwhelmed and that the cerebral hypoperfusion is being responsible for acute anoxic-ischemic brain damage. Lotgering et al. show a loss of cerebral auto-regulation from 4 min of total cord occlusion in fetuses close to term, in relation to arterial hypotension^13^. We can suspect that the same phenomenon occurs when short intermittent occlusions are continued overtime, particularly when the occlusions are very frequent, as in the severe phase of our protocol. De Haan et al. reported a decrease in ECOG signal amplitude parallel with an increase in cortical impedance reflecting a cytotoxic oedema that persisted at the beginning of the recovery phase^37^.

The global decrease in amplitude and SEF correlated with acidosis, mainly respiratory. The direct association between fetal EEG signal suppression and severe acidosis has been reported previously in the fetus^52^. The EEG signal alteration described could be directly related to a deleterious effect of acidosis on the brain. On the contrary, some recent studies have shown that hypercapnia decreases neuronal excitability with a neuroprotective effect^53–55^.

Besides the severity of the mechanism of anoxic-ischemia, the constitution of ischemic brain lesions thus appears to depend on the adaptive response of the fetus and its capacity to maintain cerebral energy requirements in the acute phase. We have noted inter-individual variability in cerebral responses to occlusion, also reported in previous studies^15,37,56^. During prolonged carotid occlusions (30 min) in Fraser’ study, some fetuses showed only a slight decrease in ECOG amplitude and SEF while others showed profound signal suppression during the occlusion^32^. This inter-individual variable cerebral response appears to be primarily related to variable fetal cardiovascular adaptability^15,37,56^. Previous mild chronic hypoxia appears to be particularly deleterious to adaptation capacity to an added acute anoxia. More rapid and profound acidosis, earlier arterial hypotension and greater suppression of EEG activity have been observed in the lamb fetus as well as reduced carotid flow and cerebral oxygen delivery, greater increase in cortical impedance and greater neuronal loss^15,35,36,57^. The “reservoir” of adaptive capacity of each fetus therefore depends not only on the cause and the mechanism of acute anoxia but also on many factors such as fetal maturation, fetal weight, multiple pregnancy, prior chronic hypoxia, aerobic reserve or maternal temperature^58^. Finally, the challenge is not only to recognise fetuses exposed to acute hypoxia during delivery but also to detect those whose adaptive capacities become insufficient. In this objective, the fetal EEG seems to be particularly informative, allowing a continuous monitoring of fetal brain activity.

In contrast to previous studies in fetal sheep, we visually analysed the raw EEG in addition to the quantitative analysis of the signal. Visual analysis guides the interpretation of the quantitative analyses by relating it to a physiological basis. This avoids misinterpretation that can occur when quantitative results are blindly analysed. For example, as explained above, an increase in SEF above 25 Hz may reflect signal suppression with an isoelectric tracing^39^, but without raw EEG visual analysis this increase may be interpreted as a simple acceleration in signal frequency. We used for this study quantitative EEG markers that have been shown to be correlated with post anoxia EEGs in humans^59^. BSR is of particular interest and had never been used before in fetal sheep EEG studies to our best knowledge. This marker appears to be more sensitive than the amplitude in reflecting periods of signal suppression, as its value is less affected by the intermittent presence of artefactual large slow waves occurring during the occasional movements of the ewe.

Limits are inherent to experimental studies with a limited number of subjects and numerous artefacts due to the technical procedures. We aimed to use a protocol that tries to mimic the different phases of labour in human. Moreover, we do not have objective markers of fetal brain lesions that could be provided by pathological analysis.

## Conclusion

In this study, the direct cerebral response of lamb fetuses to progressive anoxia was analysed using a recently developed in utero EEG recording technique. Visual analysis of the EEG was first performed to guide and interpret the quantitative analysis of the tracings. We hypothesize that EEG signal suppression during cord occlusion could reflect a cerebral adaptation mechanism that may have a neuroprotective role. The progressive alteration of the EEG signal with increasing occlusion frequency would correspond to the failure of cardiovascular adaptation mechanisms and the onset of neurological repercussions of cerebral hypoperfusion. These qEEG markers could be used to predict in real time the failure of the fetal adaptive mechanisms to anoxo-ischemia and thus the risk of HIE. These preliminary results need to be confirmed by further studies and an intrapartum EEG recording technique in the human fetus remains to be developed before a practically usable fetal brain function monitoring tool can be devised.

## Data Availability

The data that support the findings of this study are available from the corresponding author, LL, upon reasonable request.
